# Association between vaccination status and neurodevelopmental outcomes in children with cerebral palsy

**DOI:** 10.3389/fped.2026.1751777

**Published:** 2026-04-01

**Authors:** Hong Zhao, Mingbo Hu, Fei Xie, Chunyu Zhang, Linli Zhang, Chao Bai, Junjie Wu, Baofeng Yan, Aikebaier Halike, Jingxuan Xu, Xinping Luan

**Affiliations:** 1 Cerebral Palsy Center in Neurosurgery, Second Affiliated Hospital of Xinjiang Medical University, Urumqi, China; 2 Department of Neurosurgery, The First Affiliated Hospital of Henan University of Chinese Medicine, Weihui City, China; 3 Department of Neurology, The First Affiliated Hospital of Henan University of Chinese Medicine, Weihui City, China

**Keywords:** bidirectional cohort study, cerebral palsy, communication function, immunization schedule, motor function, vaccination

## Abstract

**Background:**

The safety of vaccines and their impact on functional outcomes in children with cerebral palsy remain significant concerns for both parents and healthcare providers. These concerns have limited the full implementation of routine immunization schedules in this specific population.

**Objective:**

This study aimed to investigate the association between vaccination status and the development of motor and communication functions in children with cerebral palsy. The findings are intended to inform the development of targeted vaccination strategies.

**Methods:**

We conducted a bidirectional cohort study involving 484 children diagnosed with cerebral palsy at the Second Affiliated Hospital of Xinjiang Medical University between January 2018 and December 2024. Participants were divided into a retrospective cohort (diagnosed 2018–2020, *n* = 277) and a prospective cohort (diagnosed 2021–2022, *n* = 207). Based on vaccination status, they were further classified into a vaccinated group (received at least one dose) and an unvaccinated group. Functional abilities were assessed using established classification systems, including the Gross Motor Function Classification System (GMFCS) and the Communication Function Classification System (CFCS). Vaccination completion rates were calculated according to the 2021 Chinese National Immunization Program Schedule. Statistical analyses included the Mann–Whitney U test and Spearman correlation to compare groups and examine relationships between vaccination completion rates and functional scores.

**Results:**

Children in the vaccinated group demonstrated significantly better motor function, reflected by lower GMFCS levels (*z* = 3.26, *p* = 0.001), and significantly better communication function, reflected by higher CFCS levels (*z* = 2.89, *p* = 0.004), compared to the unvaccinated group. A higher vaccination completion rate was negatively correlated with GMFCS levels (*r* = −0.24, *p* < 0.01) and positively correlated with CFCS levels (*r* = 0.22, *p* < 0.01). No significant differences were observed between the two groups in manual ability (MACS) or eating and drinking ability (EDACS). In the prospective cohort, the vaccinated group demonstrated significantly greater improvement in GMFCS and CFCS classifications during follow-up (*p* < 0.05).

**Conclusion:**

For children with cerebral palsy, routine vaccination is not only safe but may also exert a positive regulatory effect on neurological development. We recommend reinforcing routine immunization in this population and optimizing vaccination strategies through ongoing dynamic follow-up.

## 1 Introduction

Vaccines, developed since the 19th century, have been instrumental in controlling infectious diseases and saving countless lives (1). However, as the healthcare needs of specific child populations gain attention, the safety of vaccination in children with neurological conditions has become a key research focus. This is particularly relevant for children with cerebral palsy (CP), a leading cause of childhood disability. Children with CP often present with multi-system dysfunctions, including motor and speech impairments. The decision of whether to vaccinate them remains a subject of ongoing debate (2, 3).

In China, the prevalence of cerebral palsy is approximately 3‰. Certain remote areas of Xinjiang, constrained by underdeveloped economies and limited medical resources, report a significantly higher prevalence than the national average (4). Correspondingly, childhood vaccination rates in Xinjiang also lag behind those in more economically developed regions of China (5). This situation presents a critical challenge for children with CP. These children often have lower immunity and face a higher risk of threatening infectious diseases (6). Despite this increased vulnerability, caregivers often decline vaccination due to concerns about potential adverse effects on neurological function (7, 8). This dilemma primarily stems from a lack of high-quality empirical studies providing clear guidance.

To address this research gap, our study innovatively adopts a bidirectional cohort design. This approach integrates retrospective data with prospective follow-up. It aims to provide a more comprehensive understanding of the temporal relationship between vaccination and functional outcomes in children with CP. Ultimately, we intend for our findings to offer a scientific basis to support clinical practice.

## 2 Materials and methods

### 2.1 Study participants

This study included children with CP diagnosed at the Second Affiliated Hospital of Xinjiang Medical University between 1 January 2018 and 31 December 2024. Participants were divided into two cohorts based on their diagnosis date:

A retrospective cohort consisted of children diagnosed from 1 January 2018 to 31 December 2020.

A prospective cohort included children diagnosed from 1 January 2021 to 31 December 2022. These participants were followed up every six months until the study conclusion in 31/12/2024.

The inclusion criteria were: (1) meeting internationally recognized diagnostic criteria for CP; (2) The age range is 3–18 years (both age at enrollment and age at study completion must fall within this range); (3) guardian provision of signed informed consent. Exclusion criteria were: (1) previous neurosurgical intervention; (2) co-existing neurological disorders such as hydrocephalus, stroke, or meningitis; (3) conditions affecting motor function like fractures or polio; (4) organic brain lesions related to language function; (5) contraindications to vaccination, such as refractory epilepsy (According to the Childhood Immunization Schedule for National Immunization Program Vaccines—China, Version 2021) (9); (6) children born to HIV-positive mothers.

Sample size calculations were based on pre-trial data using G*Power 3.1 software, with a medium effect size (*d* = 0.3), *α* = 0.05, and test power = 0.80. This yielded a minimum sample size requirement of 350 cases. Accounting for dropouts and subgroup analyses, 484 cases were ultimately included. Among these: The retrospective cohort contained 277 children (178 in the vaccinated group, 99 in the unvaccinated group). The prospective cohort contained 207 children (160 in the vaccinated group, 47 in the unvaccinated group). Baseline characteristics, including age, gender, and CP type (spastic/mixed/dyskinetic), showed no statistically significant differences between the cohorts (*p* > 0.05, Table 1). This study received approval from the hospital's Medical Ethics Committee (Approval No: KY2024050907).

**Table 1:** Baseline data statistics.

		Exposure group			Non-exposed group			F/*χ*²/Z	*p*
Baseline characteristics		Follow-up group	Retrospective group	Total	Follow-up group	Retrospective group	Total		
		160	178	338	47	99	146		
Age (years)		7.28 ± 3.93	10.62 ± 3.37	9.03 ± 4.00	7.17 ± 3.52	9.07 ± 4.39	8.46 ± 4.20	1.81	0.179
Gender, n (%)									
	Male	103	105	208 (61.5%)	26	55	81 (55.5%)		
	Female	57	73	130 (38.5%)	21	44	65 (44.5%)	1.556	0.21
Vaccination rate (%)		59% (45%, 73%)	55% (45%, 64%)	NA	NA	NA	NA	3.930	0.45
Co-occurring epilepsy, n (%)									
	Yes	19	34	53 (15.9%)	6	11	17 (11.6%)		
	No	141	144	285 (84.3%)	41	88	129 (88.4%)	1.343	0.24
Classification, n (%)									
	Spastic type	93	91	184 (54.4%)	18	57	75 (51.3%)		
	Athetoid type	11	15	26 (7.6%)	5	5	10 (6.8%)		
	Mixed type	56	72	128 (37.8%)	24	37	61 (41.8%)	0.676	0.713
GMFCS, n (%)									
	I	20	7	27 (7.9%)	6	22	28 (19.1%)		
	II	53	28	81 (23.9%)	9	22	31 (21.2%)		
	III	29	52	81 (23.9%)	11	24	35 (23.9%)		
	IV	17	51	68 (20.1%)	8	16	24 (16.4%)		
	V	41	40	81 (23.9%)	13	15	28 (19.1%)	−2.41	0.016
CFCS, n (%)									
	O	10	6	16 (4.7%)	9	5	14 (9.5%)		
	I	26	31	57 (16.8%)	18	14	32 (21.9%)		
	II	58	72	130 (38.4%)	12	45	57 (39.0%)		
	III	33	61	94 (27.8%)	6	22	28 (19.1%)		
	IV	33	8	41 (12.1%)	2	13	15 (10.2%)	−2.593	0.01
MACS, n (%)									
	I	19	5	24 (7.1%)	16	1	12 (8.2%)		
	II	49	37	86 (25.4%)	9	10	51 (34.9%)		
	III	36	65	101 (29.8%)	15	21	37 (25.3%)		
	IV	33	42	75 (22.1%)	5	53	19 (13.0%)		
	V	23	29	52 (15.3%)	2	14	27 (18.4%)	−1.712	0.087
EDACS, n (%)									
	I	27	25	52 (15.3%)	8	9	17 (9.5%)		
	II	72	71	143 (42.2%)	16	44	60 (34.9%)		
	III	43	64	107 (31.6%)	16	27	43 (28.1%)		
	IV	16	18	34 (10.0%)	5	14	19 (17.8%)		
	V	2	0	2 (0.6%)	2	5	7 (9.5%)	−1.48	0.139

### 2.2 Assessment methods

Functional status was evaluated using internationally recognized classification systems (10). Assessments were performed independently by two rehabilitation physicians, each with over five years of experience. Inter-rater reliability was assessed using Cohen's Kappa coefficient for categorical classifications (GMFCS, CFCS, MACS, EDACS). The Kappa value exceeded 0.85, indicating excellent agreement between the two raters.

Motor function was assessed using the Gross Motor Function Classification System (GMFCS, 2007 edition). This system classifies function into Levels I to V, where Level I represents the best function and Level V the worst. It is applicable for children aged 2–18 years.

Communication function was evaluated using the Communication Function Classification System (CFCS). This system ranges from Level I (most effective communicator) to Level V (least effective communicator), for children aged 2–18 years.

Manual ability was classified using the Manual Ability Classification System (MACS) for children with CP. Levels range from I (handles objects easily) to V (does not handle objects). For children aged 3–4 years, assessment is conducted using the Mini-MACS, which is suitable for younger children; For children aged ≥4 years, the Modified Apoplectic Cerebral palsy Hand Function Scale (MACS) is employed.

Eating and drinking ability was assessed using the Eating and Drinking Ability Classification System (EDACS). This system classifies ability from Level I (eats and drinks safely) to Level V (requires tube feeding). It is suitable for children aged 3 years and above.

### 2.3 Study procedures

#### 2.3.1 Data collection

For the retrospective cohort, initial functional assessment data (GMFCS, CFCS, MACS, EDACS) at diagnosis were extracted from the hospital's electronic medical records. Guardians were contacted via telephone to supplement vaccination records, including reasons for non-vaccination. The functional assessment results at the last visit were collected.

For the prospective cohort, baseline functional assessments were conducted upon enrollment, and follow-up files were established. Vaccination data, including timing, doses, and vaccine types, were recorded during outpatient reviews or telephone follow-ups. Functional assessments were repeated every 6 months to document any changes in classification levels.

#### 2.3.2 Exposure and outcome definitions

The primary exposure factor was vaccination status, categorizing participants into a vaccinated (exposed) group and an unvaccinated (non-exposed) group (11). Vaccination completion rate was calculated using the formula: Vaccination Completion Rate = (Number of vaccine doses received at time of assessment/Recommended number of doses for age at time of assessment) × 100%. Recommendations were based on the “2021 Chinese National Immunization Program Schedule for Children” (9).

The primary outcome measures were statistically significant differences in GMFCS, CFCS, MACS, and EDACS levels between the exposed and non-exposed groups. Functional changes during follow-up were also considered outcomes. A decrease in GMFCS, MACS, or EDACS level indicated functional improvement. An increase in CFCS level indicated improvement in communication ability.

#### 2.3.3 Statistical analysis

Data were analyzed using SPSS software (version 27.0). Normality of continuous variables was assessed visually using histograms and probability plots, and analytically using the Kolmogorov–Smirnov test for samples larger than 50.Variables conforming to continuous normal distribution were expressed as mean ± standard deviation, with intergroup comparisons conducted using t-tests; non-normally distributed variables were presented as median (interquartile range, IQR), analyzed using the Mann–Whitney U test; categorical variables were assessed using *χ*²tests; ordinal data were evaluated using the Mann–Whitney U test and Spearman correlation analysis. Partial correlation analysis was employed to adjust for potential confounding factors, such as age, CP type, and family socioeconomic status. A statistically significant difference is indicated when *p* < 0.05.

## 3 Results

### 3.1 Comparison of baseline characteristics

The vaccinated group and the unvaccinated group were comparable in terms of age, gender, presence of comorbid epilepsy, and CP type. The vaccination rates between the prospective and retrospective cohorts within the vaccinated group showed no statistically significant difference (Table 1).

### 3.2 Distribution of reasons for vaccination delay

**Figure 1:**
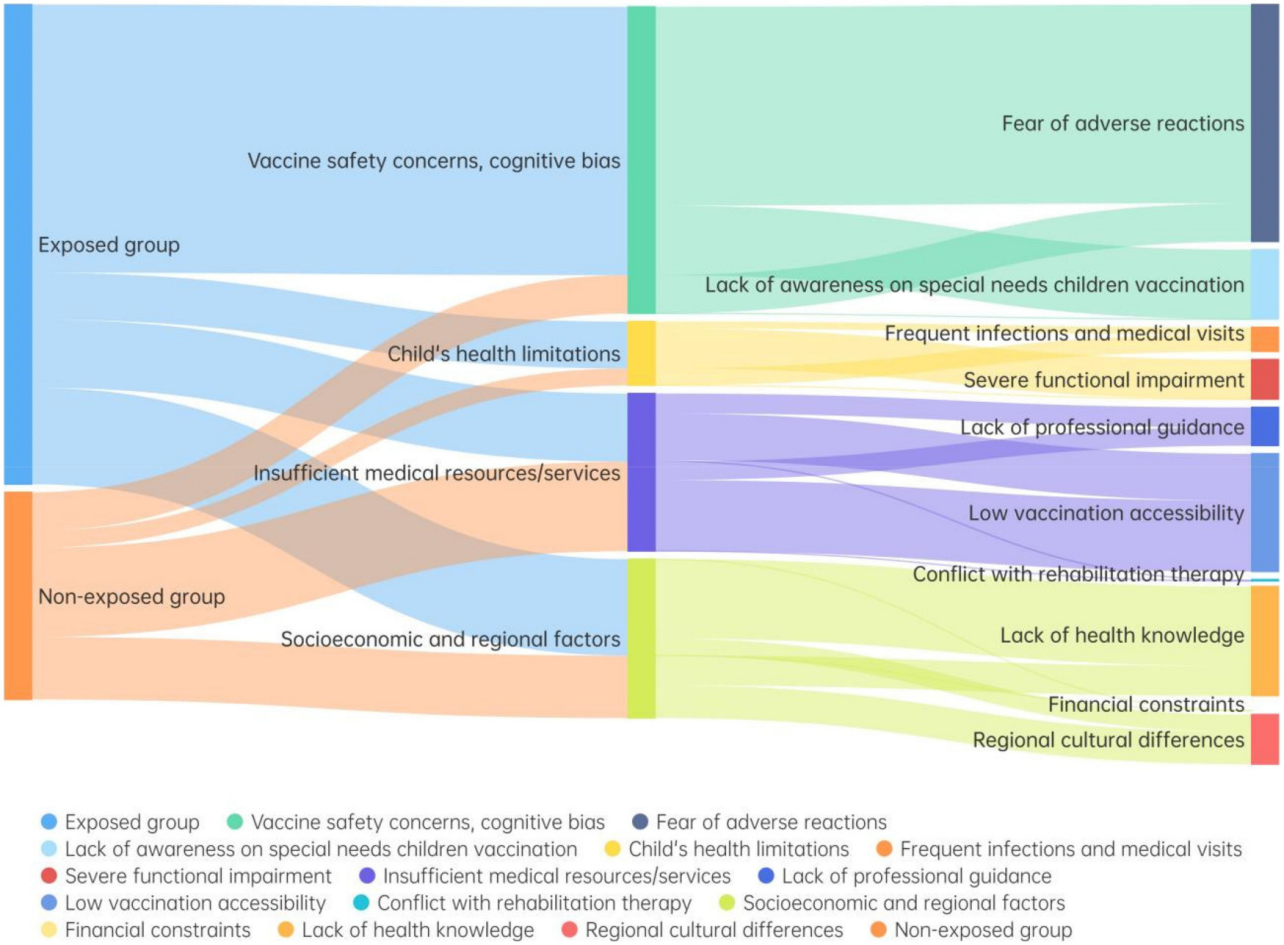
Sankey diagram of reasons for delayed or unvaccinated children with cerebral palsy. The diagram categorizes reasons into four major types (safety concerns, medical resource limitations, cultural differences, and misconceptions) and 11 specific factors. Width of flows represents the proportion of children reporting each reason, stratified by vaccination status (vaccinated vs. unvaccinated). Vac, vaccinated; Unvac, unvaccinated.

The raw data analysis showed that there was a delay in vaccination or no vaccination in 484 children, including 338 children in the Vaccinated group and 146 children in the Unvaccinated group (Table 2). The reasons for these delays were categorized into 4 major types, encompassing 11 specific factors. A Sankey diagram was generated to visually represent this data (Figure 1).

**Table 2:** Distribution of reasons for delayed vaccination in children with cerebral palsy (n, %).

Category of delay reasons	Specific reasons	Vaccinated group (*n* = 338)	Unvaccinated group (*n* = 146)	Total (*n* = 484)	Proportion (%)
Vaccine safety concerns, cognitive bias	Fear of adverse reactions	140	27	167	44.6
	Lack of awareness on special needs children vaccination	49	0	49	13.1
Child's health limitations	Frequent infections and medical visits	5	12	17	4.5
	Severe functional impairment	28	0	28	7.5
Insufficient medical resources/services	Lack of professional guidance	14	13	27	7.2
	Low vaccination accessibility	33	50	83	22.2
	Conflict with rehabilitation therapy	1	0	1	0.3
Socioeconomic and regional factors	Financial constraints	0	0	0	0
	Lack of health knowledge	56	21	77	20.6
	Regional cultural differences	12	23	35	9.4

### 3.3 Association between vaccination status and functional classifications

Overall analysis showed that children in the vaccinated group had significantly better motor function, as indicated by GMFCS levels, compared to those in the unvaccinated group (*z* = 3.26, *p* = 0.001). Their communication function, measured by CFCS levels, was also significantly better (*z* = 2.89, *p* = 0.004). However, no statistically significant differences were observed between the two groups in manual ability (MACS) or eating and drinking ability (EDACS).

### 3.4 Correlation between vaccination completion rate and functional outcomes

**Figure 2:**
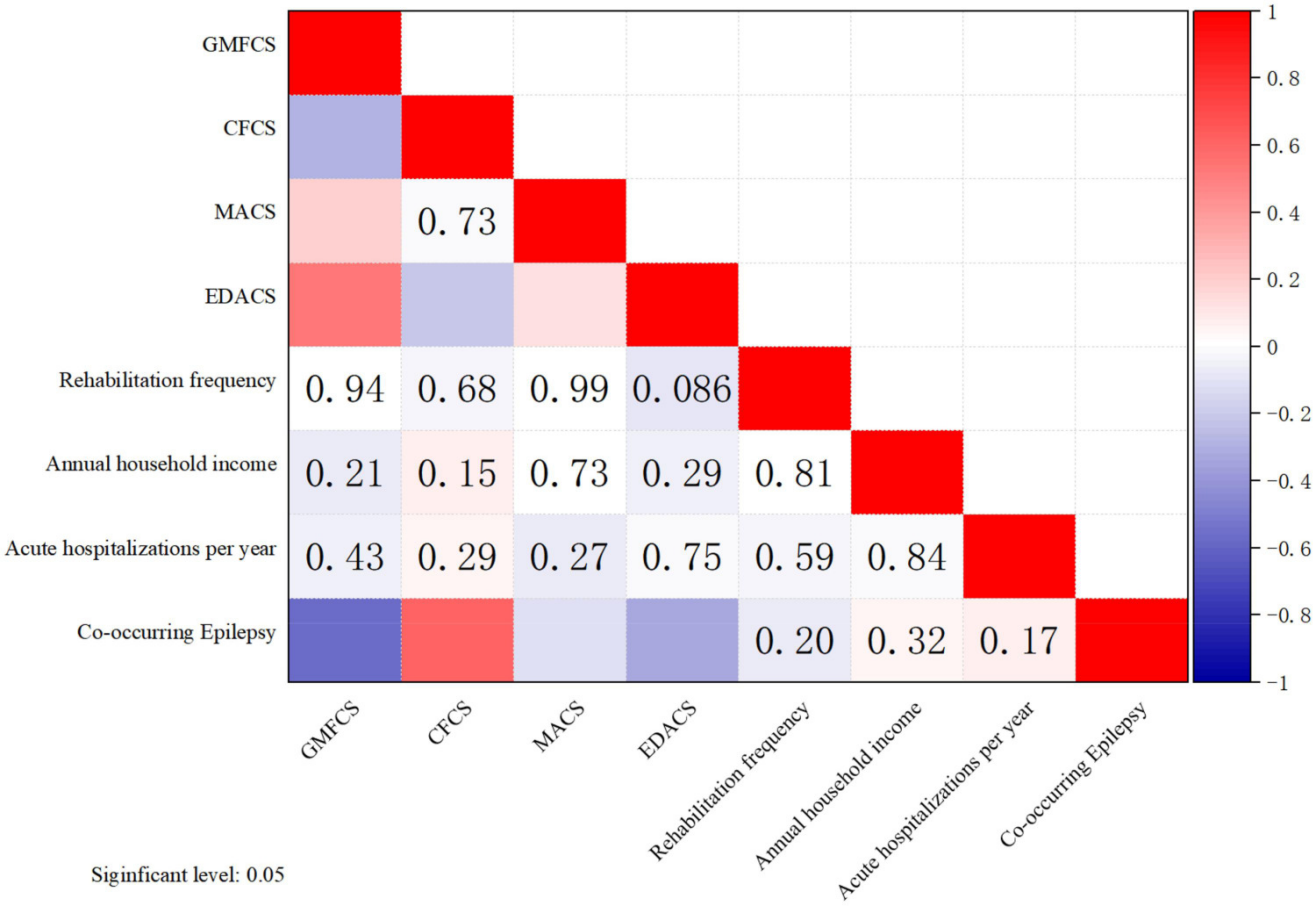
Spearman correlation analysis between vaccination completion rate and functional classification level. GMFCS, Gross Motor Function Classification System (Level I = best function, Level V = worst function); CFCS, Communication Function Classification System (Level I = least effective communicator, Level V = most effective communicator); MACS, Manual Ability Classification System (Level I = handles objects easily, Level V = does not handle objects); EDACS, Eating and Drinking Ability Classification System (Level I = eats and drinks safely, Level V = requires tube feeding).

Spearman correlation analysis indicated a negative correlation between the vaccination completion rate and GMFCS levels (*r* = −0.24, *p* < 0.01, Figure 2). A positive correlation was found between the vaccination completion rate and CFCS levels (*r* = 0.22, *p* < 0.01).Negatively correlated with MACS grading (*r* = −0.12, *p* < 0.05).Negatively correlated with EDACS grading (*r* = −0.33, *p* < 0.05).There was no significant correlation with factors such as rehabilitation frequency, the presence of concomitant controllable epilepsy, annual household income, or the number of acute infections during follow-up.

### 3.5 Comparison of functional classification improvement rates during follow-up

In a prospective cohort (*n* = 207), we performed a comparative analysis of functional improvement between the exposed group (vaccinated group, *n* = 160) and the non-exposed group (unvaccinated group, *n* = 47) over a 24-month follow-up period. Improvement was defined as a reduction of ≥1 level in GMFCS, MACS, or EDACS, or an increase of ≥1 level in CFCS from baseline to the 24-month follow-up. Between-group comparisons were conducted using the chi-square test.

The results are presented in Table 3. Regarding gross motor function, 54 children (33.7%) in the vaccinated group showed improvement in GMFCS (reduction ≥1 level), which was significantly higher than the 9 children (19.1%) in the unvaccinated group (*p* = 0.04). The median GMFCS level in the vaccinated group improved from IV (III–IV) at baseline to III (II–III) at the end of follow-up. In contrast, the unvaccinated group maintained a median level of III (II–IV) without significant change.

**Table 3:** Comparison of functional improvement rates between vaccinated and unvaccinated groups during follow-up (prospective cohort, *n* = 207).

Functional Measure	Group	Baseline level, median (IQR)	24-month follow-up median (IQR)	Patients with improvement, *n* (%)	*p*-value (between groups)
GMFCS	Vaccinated	IV (III–IV)	III (II–III)	54 (33.7)	0.04*
	Unvaccinated	III (II–IV)	III (II–IV)	9 (19.1)	
CFCS	Vaccinated	II (II–III)	II (I–III)	61 (38.1)	0.02*
	Unvaccinated	II (II–III)	II (II–III)	8 (17.0)	
MACS	Vaccinated	II (II–III)	II (II–III)	50 (31.2)	0.41
	Unvaccinated	II (II–III)	II (II–III)	15 (31.9)	
EDACS	Vaccinated	III (III–IV)	III (III–IV)	33 (20.6)	0.54
	Unvaccinated	IV (III–IV)	IV (III–IV)	10 (21.2)	

* *p* < 0.05.

In terms of communication function, 61 children (38.1%) in the vaccinated group exhibited improvement in CFCS (increase ≥1 level), also significantly higher than the 8 children (17.0%) in the unvaccinated group (*p* = 0.02). The median CFCS level in the vaccinated group improved from II (II–III) at baseline to II (I–III) at follow-up completion. The unvaccinated group, however, remained at II (II–III) without change.

Nevertheless, no statistically significant differences were observed between the two groups in improvement rates for manual ability (MACS) or eating and drinking ability (EDACS) during the follow-up period.

### 3.6 Partial correlation analysis

After controlling for variables, the partial correlation analysis results indicate (Table 4). After these adjustments, the correlation between vaccination completion rate and GMFCS levels remained significant (*r* = −0.429, *p* < 0.01). The correlation with CFCS levels also remained significant (*r* = 0.573, *p* < 0.01). There was a slight negative correlation with the frequency of rehabilitation (*r* = –0.131, *p* = 0.017). No significant correlation was found with factors such as MACS grading, EDACS grading, whether epilepsy was controllable with concomitant treatment, annual household income, or the number of acute infections during follow-up.

**Table 4:** Partial correlation analysis of vaccine completion rate in children with cerebral palsy.

	GMFCS	CFCS	MACS	EDACS	Rehabilitation frequency	Co-occurring epilepsy	Annual household income	Acute hospitalizations per year
Vaccine completion correlation coefficient	−0.434	0.574	0.001	−0.009	−0.131	0.084	−0.24	−0.04
*p*	0.01^a^	0.01^a^	0.985	0.863	0.017^b^	0.127	0.664	0.465

^a^ At the 0.01 level (two-tailed), the correlation is significant.

^b^ At the 0.05 level (two-tailed), the correlation is significant.

## 4 Discussion

This bidirectional cohort study, integrating retrospective data and prospective follow-up, provides robust evidence for the association between routine vaccination and functional outcomes in children with CP, with particular relevance to the understudied population in Xinjiang, China. Our key findings reveal that vaccinated CP children exhibited significantly better gross motor function (lower GMFCS grades) and communication abilities (higher CFCS grades) compared to their unvaccinated counterparts. Notably, the prospective cohort demonstrated that vaccination was associated with a greater likelihood of functional improvement over 24 months, with 33.7% of vaccinated children showing GMFCS improvement and 38.1% achieving CFCS enhancement—rates substantially higher than those in the unvaccinated group (19.1% and 17.0%, respectively). In contrast, no significant differences in hand function (MACS) or feeding ability (EDACS) were observed between the two groups, suggesting that the beneficial effects of vaccination are specific to whole-body coordination and higher-order cognitive-communicative functions rather than localized fine motor or swallowing skills.

Two interrelated mechanisms may explain the observed associations between vaccination and improvements in motor and communication functions. First, the infection prevention pathway is well-supported by our data and existing literature. The immune systems of children with cerebral palsy are often in a vulnerable state (6), with a significantly increased risk of infection (12). Studies have shown that pro-inflammatory cytokines such as IL-6 and TNF-α, which are elevated during infections, exacerbate neuroinflammation, impair synaptic plasticity, and disrupt the integrity of the motor cortex and Broca's area (13, 14). Vaccination directly reduces the risk of vaccine-preventable diseases (e.g., pertussis, hepatitis B), thereby decreasing the incidence of severe inflammation and its neurotoxic sequelae (15). This aligns with our observation that vaccinated children had fewer hospitalizations related to acute infections during follow-up, creating a favorable neuroprotective environment for functional recovery.

Beyond simple infection prevention, the immune modulation hypothesis offers another perspective. Vaccine adjuvants may activate innate immune pathways. This activation can promote regulatory T cell differentiation, which helps suppress excessive inflammatory responses (16). Such vaccine-induced immune homeostasis could create a systemic and central nervous system anti-inflammatory microenvironment. This environment might reduce baseline neuroinflammation unrelated to active infection. It could also indirectly support synaptic remodeling and neuronal survival through cytokine-mediated signaling (17). Particularly in brain regions critical for motor coordination and language processing. Our findings regarding the specificity of GMFCS and CFCS (as opposed to MACS or EDACS) may reflect that gross motor and communication functions rely more on extensive neural networks. Compared to localized neural pathways controlling fine motor skills and swallowing, these networks are more susceptible to systemic inflammation and exhibit more pronounced responses to immunomodulation. This provides an intrinsic biological basis for functional rehabilitation. Although this hypothesis requires further validation in the population with cerebral palsy, it offers a novel perspective for understanding the nonspecific effects of vaccines.

Our analysis of delayed vaccination reasons highlights region-specific challenges that must be addressed to optimize immunization coverage in CP children (18), especially in resource-limited areas like Xinjiang. The most prominent barriers were vaccine safety concerns (44.6% of total delays) and insufficient medical resources (22.2% citing low vaccination accessibility), consistent with global trends (19, 20) but with unique regional nuances. Notably, 60.2% of children reporting low vaccination accessibility were unvaccinated, reflecting the logistical challenges of accessing fixed vaccination sites for CP children who often require assistive devices and accompanied travel (21). Additionally, regional cultural differences accounted for 15.8% of delays in the unvaccinated group—nearly five times higher than in the vaccinated group—indicating lower trust in modern medical interventions among certain ethnic minority families in Xinjiang. The unique finding of 13.1% of parents misunderstanding that “special children have limited social contact and do not need vaccination” (exclusively in the vaccinated group) suggests that unvaccinated parents hold more entrenched safety concerns, while vaccinated parents may have residual doubts about vaccination necessity.

These barriers are exacerbated by socioeconomic factors and nomadic lifestyles among certain ethnic minority groups in Xinjiang, disrupting the conventional vaccination schedule—challenges similar to those faced by migrant families in India (22). Additionally, there is a moderate but significant negative correlation between vaccination completion rates and recovery frequency (*r* = −0.131, *p* = 0.017), which may be attributed to scheduling conflicts between vaccination and recovery. Addressing these issues requires context-specific strategies: expanding mobile vaccination services to remote areas, developing multilingual health education materials to address cultural and cognitive barriers, and integrating vaccination reminders with routine recovery visits to minimize scheduling conflicts.

It must be acknowledged that vaccination may serve as a surrogate indicator for family health-seeking behaviors. Vaccinated children are more likely to come from families with abundant medical resources or heightened health awareness, which often exhibit greater engagement in rehabilitation training, nutritional interventions, and early education—factors that may collectively promote functional improvement (23, 24). In underdeveloped regions such as Xinjiang, low vaccination rates are frequently associated with the absence of rehabilitation services and insufficient health knowledge among parents (25). Unvaccinated children may simultaneously face multiple adverse conditions, including poverty, poor transportation access, and a lack of rehabilitation facilities, leading to poorer functional outcomes. Although this study adjusted for key confounding factors such as age, type of cerebral palsy, and family income through partial correlation analysis, unmeasured variables (e.g., rehabilitation intensity, parental education level, and quality of home care) may have influenced the results, posing a risk of overestimating the direct effects of vaccination.

## 5 Limitations

This study also has several limitations: First, the single-center design may introduce selection bias, as all participants were from the Second Affiliated Hospital of Xinjiang Medical University, and the results need to be validated in multicenter studies across different geographical and socioeconomic backgrounds. Second, the study did not differentiate between inactivated vaccines and live attenuated vaccines, nor did it analyze the functional differences of specific vaccine types (e.g., DPT, MMR vaccines). Finally, the 24-month follow-up period was insufficient to assess the long-term trajectory of neurodevelopment, and longer-term studies are required.

## 6 Conclusion

In conclusion, this bidirectional cohort study demonstrates that routine vaccination is safe and associated with improved gross motor and communication functions in children with cerebral palsy, with more pronounced functional gains observed in vaccinated children during prospective follow-up. Key barriers to vaccination in Xinjiang include safety concerns, limited accessibility, and cultural differences, which require targeted interventions to enhance immunization coverage. Clinicians should actively promote routine vaccination for CP children, supplemented by personalized health education to address parental hesitancy. Future multi-center, long-term studies are needed to validate these findings, explore vaccine-specific effects, and optimize immunization strategies for this vulnerable population.

## Data Availability

The raw data supporting the conclusions of this article will be made available by the authors, without undue reservation.

## References

[1] Dubé E Laberge C Guay M Bramadat P Roy R Bettinger J. Vaccine hesitancy: an overview. Hum Vaccin Immunother. (2013) 9(8):1763–73. 10.4161/hv.24657 23584253 PMC3906279

[2] Liba Z Kraus J Necas T Necas J Klugar M Krsek P. Movement disorders, cerebral palsy and vaccination. Eur J Paediatr Neurol. (2022) 36:143–50. 10.1016/j.ejpn.2021.12.006 34979476

[3] Jahan I Vakaloloma U Perera S Tuibeqa I Devi R Volavola L Vaccination and its social and behavioural drivers in children with disability in Fiji. BMJ Glob Health. (2025) 10(5):e017510. 10.1136/bmjgh-2024-017510 40345704 PMC12067796

[4] Wu J Bai C Hu M Guan Q Li J Luan X Efficacy of cervical perivascular sympathectomy in improving upper limb motor function in children with cerebral palsy and construction of a predictive model. Clin Neurol Neurosurg. (2024) 240:108273. 10.1016/j.clineuro.2024.108273 38608351

[5] Zhang H Lai X Mak J Sriudomporn S Zhang H Fang H Coverage and equity of childhood vaccines in China. JAMA Netw Open. (2022) 5(12):e2246005. 10.1001/jamanetworkopen.2022.46005 36484985 PMC9856225

[6] Khuc THH Karim T Cao MC Nguyen TVA Nguyen THG Trinh QD Immunization status of children with cerebral palsy: a cross-sectional hospital-based study in Vietnam. PLoS One. (2025) 20(5):e0323081. 10.1371/journal.pone.0323081 40333874 PMC12057921

[7] Ghazy RM Sallam M Fadl N Bouraad E Youssef N Ghoneim OSA. Attitude of parents of children with cerebral palsy towards COVID-19 vaccination. Int J Environ Res Public Health. (2023) 20(3):1909. 10.3390/ijerph20031909 36767281 PMC9915268

[8] Wang Q Yang L Li L Xiu S Yang M Wang X Investigating parental perceptions of respiratory syncytial virus (RSV) and attitudes to RSV vaccine in Jiangsu, China: insights from a cross-section study. Vaccine. (2025) 44:126570. 10.1016/j.vaccine.2024.126570 39612804

[9] Childhood immunization schedule for National Immunization Program Vaccines—China (Version 2021). 10.46234/ccdcw2021.270 PMC8855077 35186365

[10] Paulson A Vargus-Adams J. Overview of four functional classification systems commonly used in cerebral palsy. Children. (2017) 4(4):30. 10.3390/children4040030 28441773 PMC5406689

[11] Nordström P Ballin M Nordström A. Safety and effectiveness of monovalent COVID-19 mRNA vaccination and risk factors for hospitalisation caused by the omicron variant in 0.8 million adolescents: a nationwide cohort study in Sweden. PLoS Med. (2023) 20(2):e1004127. 10.1371/journal.pmed.1004127 36802397 PMC9990916

[12] Jahan I Al Imam MH Karim T Muhit M Hardianto D Das MC Epidemiology of cerebral palsy in Sumba Island, Indonesia. Dev Med Child Neurol. (2020) 62(12):1414–22. 10.1111/dmcn.14616 32686098

[13] Hu M Bai C Zhao H Wu J Luan X. Research progress on the role of the interleukin family in the pathogenesis of cerebral palsy in children. J Integr Neurosci. (2024) 23(12):213. 10.31083/j.jin2312213 39735959

[14] Tervo RC Taylor B. Vaccinations and the physically handicapped child. Can Med Assoc J. (1982) 127(6):475–7. 6214304 PMC1862073

[15] Greenwood VJ Crawford NW Walstab JE Reddihough DS. Immunisation coverage in children with cerebral palsy compared with the general population. J Paediatr Child Health. (2013) 49(2):E137–41. 10.1111/jpc.12097 23360148

[16] Bayry J. Regulatory T cells as adjuvant target for enhancing the viral disease vaccine efficacy. Virusdisease. (2014) 25(1):18–25. 10.1007/s13337-013-0187-3 24426307 PMC3889236

[17] Batista-Duharte A Téllez-Martínez D Fuentes DLP Carlos IZ. Molecular adjuvants that modulate regulatory T cell function in vaccination: a critical appraisal. Pharmacol Res. (2018) 129:237–50. 10.1016/j.phrs.2017.11.026 29175113

[18] Yang L Peng J Deng J He F Chen C Yin F Vaccination Status of children with epilepsy or cerebral palsy in Hunan rural area and a relative KAP survey of vaccinators. Front Pediatr. (2019) 7:84. 10.3389/fped.2019.00084 30984716 PMC6448507

[19] Han Y Wang Q Zhao S Wang J Dong S Cui T Parental category B vaccine hesitancy and associated factors in China: an online cross-sectional survey. Expert Rev Vaccines. (2022) 21(1):145–53. 10.1080/14760584.2022.2008247 34792433

[20] Feng T Wang X Li J Wang C Qiu Y Zhang Y Common issues and improvement solution of vaccine hesitancy in children with underlying neurological conditions: experience from one national children’s medical center in China. Vaccine. (2023) 41(2):427–34. 10.1016/j.vaccine.2022.11.063 36470687

[21] Wiart L Darrah J Kembhavi G. Stretching with children with cerebral palsy: what do we know and where are we going? Pediatr Phys Ther. (2008) 20(2):173–8. 10.1097/PEP.0b013e3181728a8c 18480717

[22] Priya PK Pathak VK Giri AK. Vaccination coverage and vaccine hesitancy among vulnerable population of India. Hum Vaccin Immunother. (2020) 16(7):1502–7. 10.1080/21645515.2019.1708164 32017653 PMC7482787

[23] Novak I Morgan C Adde L Blackman J Boyd RN Brunstrom-Hernandez J Early, accurate diagnosis and early intervention in cerebral palsy. JAMA Pediatr. (2017) 171(9):897–907. 10.1001/jamapediatrics.2017.1689 28715518 PMC9641643

[24] Richards CL Malouin F. Cerebral palsy: definition, assessment and rehabilitation. Handb Clin Neurol. (2013) 111:183–95. 10.1016/B978-0-444-52891-9.00018-X 23622163

[25] May P Smithers-Sheedy H Muhit M Cumming R Jones C Booy R Immunisation status of children with cerebral palsy in rural Bangladesh: results from the Bangladesh cerebral palsy register (BCPR). Infect Disord Drug Targets. (2020) 20(3):318–22. 10.2174/1871526518666181024101002 30360749

